# 1650. Efficacy of β-Lactam Therapy against Infections Caused by Metallo-β-lactamase (MBL)-Producing Enterobacterales: Bridging the Susceptibility Testing Gap

**DOI:** 10.1093/ofid/ofac492.116

**Published:** 2022-12-15

**Authors:** Kamilia Abdelraouf, Christian M Gill, Matthew Gethers, Giusy Tiseo, Marco Falcone, Francesco Menichetti, David P Nicolau

**Affiliations:** Hartford Hospital, Hartofrd, CT; Hartford Hospital, Hartofrd, CT; Hartford Hospital, Hartofrd, CT; Infectious Diseases Unit/University of Pisa, Pisa, Toscana, Italy; Infectious Diseases Unit/University of Pisa, Pisa, Toscana, Italy; Department of Clinical and Experimental Medicine, Infectious Diseases Unit, University of Pisa, Pisa, Italy, Pisa, Toscana, Italy; Hartford Hospital, Hartofrd, CT

## Abstract

**Background:**

*In vitro-in vivo* discordance in the activity of β-lactams against MBL-producing Enterobacterales has been described. This discordance is likely attributed to the supra-physiologic zinc level in the *in vitro* testing media, which facilitates the bicyclic β-lactam ring hydrolysis. In this study, we compared the outcome of empirical non-MBL-active β-lactam therapy (carbapenems and ceftazidime/avibactam) and MBL-active β-lactam therapy (ceftazidime/avibactam plus aztreonam) among patients with bloodstream infections due to NDM-producing *Klebsiella pneumoniae*. Validation of the efficacy of carbapenem in a murine septicemia model was conducted. *In vitro* susceptibility testing conditions were altered to better predict the *in vivo* outcome.

**Methods:**

A retrospective observational study of patients admitted to hospitals in Italy. The primary outcome was 14-day all-cause mortality. Cox regression analysis was performed to evaluate primary outcome. Kaplan Meier survival and log-rank test were used to compare 14-day mortality between patient’s cohorts. Septicemia was induced in mice via intraperitoneal inoculation with the isolates retrieved from the patients then clinical exposure of meropenem (MEM; 2 g q8h 3h infusion) was given for 2 days. Survival was recorded for 4 days and compared with sham controls. Unbound zinc levels were measured in human and infected mice plasma. MEM MICs were determined in Mueller Hinton Broth (MHB) and MHB adjusted to the physiologic zinc levels.

**Results:**

Of the patients identified, 29 received empirical non-MBL-active β-lactams for median duration 4 days while 29 received MBL-active β-lactams. The 14-day mortality rate was 21% in the non-MBL-active group vs 14% in the MBL-active group (P = 0.73) and survival patterns were not significantly different (Fig. 1). Cox regression showed that use of non-MBL-active therapy was not associated with significantly increased 14-day mortality (hazard ratio = 1.45; P = 0.57). MEM treatment resulted in protection from mortality in mice (Fig. 2). MEM MICs in zinc-adjusted MHB were 1- to > 16-fold lowered relative to MICs in MHB ( ≥ 64 mg/L).

**Conclusion:**

Our data provide foundational support to help establish PK/PD relationships using MICs derived in physiologic zinc levels which may better predict β-lactam therapy outcome.

**Disclosures:**

**Kamilia Abdelraouf, PhD**, Evopoint Biosciences Co., Ltd: Grant/Research Support|Venatorx Pharmaceuticals, Inc.: Grant/Research Support **Christian M. Gill, PharmD**, Everest Medicines, Shionogi, Cepheid: Grant/Research Support **Matthew Gethers, PhD**, Thermo Fisher: Employee **Marco Falcone, MD, PhD**, GILEAD: Grant/Research Support|GSK: Honoraria|MENARINI: Advisor/Consultant|MENARINI: Grant/Research Support|MSD: Honoraria|PFIZER: Honoraria|SHIONOGI: Grant/Research Support **Francesco Menichetti, n/a**, Aneglini: Advisor/Consultant|Aneglini: Board Member|Aneglini: Grant/Research Support|Aneglini: Honoraria|Astellas: Advisor/Consultant|Astellas: Honoraria|Becton: Advisor/Consultant|Becton: Honoraria|bioMérieux: Advisor/Consultant|bioMérieux: Honoraria|Biotest: Advisor/Consultant|Biotest: Board Member|Biotest: Honoraria|Bristol-Myers Squibb: Advisor/Consultant|Bristol-Myers Squibb: Honoraria|Correvio: Advisor/Consultant|Correvio: Speaker honoraria|Dickinson: Advisor/Consultant|Dickinson: Honoraria|Gilead: Advisor/Consultant|Gilead: Grant/Research Support|Janssen: Advisor/Consultant|Janssen: Honoraria|MSD: Advisor/Consultant|MSD: Speaker honoraria|Nordic pharma: Board Member|Nordic pharma: Honoraria|Pfizer: Advisor/Consultant|Pfizer: Honoraria|Shionogi: Advisor/Consultant|Shionogi: Honoraria|ViiV: Advisor/Consultant|ViiV: Honoraria **David P. Nicolau, PharmD**, Shionogi: Grant/Research Support.

